# Marine Compounds with Therapeutic Potential in Gram-Negative Sepsis

**DOI:** 10.3390/md11062216

**Published:** 2013-06-19

**Authors:** Tamara Solov’eva, Viktoria Davydova, Inna Krasikova, Irina Yermak

**Affiliations:** G.B. Elyakov Pacific Institute of Bioorganic Chemistry, Far-Eastern Branch, the Russian Academy of Sciences, pr. 100 let Vladivostoku, 159, Vladivostok 690022, Russia; E-Mails: soltaf@mail.ru (T.S.); innakras@mail.ru (I.K.); iren@piboc.dvo.ru (I.Y.)

**Keywords:** sepsis, endotoxin, endotoxin antagonists, lipid A, chitosan, carrageenan

## Abstract

This paper concerns the potential use of compounds, including lipid A, chitosan, and carrageenan, from marine sources as agents for treating endotoxemic complications from Gram-negative infections, such as sepsis and endotoxic shock. Lipid A, which can be isolated from various species of marine bacteria, is a potential antagonist of bacterial endotoxins (lipopolysaccharide (LPSs)). Chitosan is a widespread marine polysaccharide that is derived from chitin, the major component of crustacean shells. The potential of chitosan as an LPS-binding and endotoxin-neutralizing agent is also examined in this paper, including a discussion on the generation of hydrophobic chitosan derivatives to increase the binding affinity of chitosan to LPS. In addition, the ability of carrageenan, which is the polysaccharide of red alga, to decrease the toxicity of LPS is discussed. We also review data obtained using animal models that demonstrate the potency of carrageenan and chitosan as antiendotoxin agents.

## 1. Introduction

Gram-negative bacterial sepsis and its extreme manifestation, septic (endotoxic) shock, continue to be a leading cause of death among hospitalized patients [1]. The incidence of sepsis and its associated mortality are increasing with the growing use of invasive procedures and both immunosuppressive and cytotoxic therapies in medical practice, as well as the aging of the population, which is accompanied by an increase in chronic diseases. The development of new antibiotics and their introduction into clinical practices do not fundamentally overcome this problem because treating septic patients with antibiotics may in fact cause the patients’ health to worsen rather than improve.

**Figure 1:**
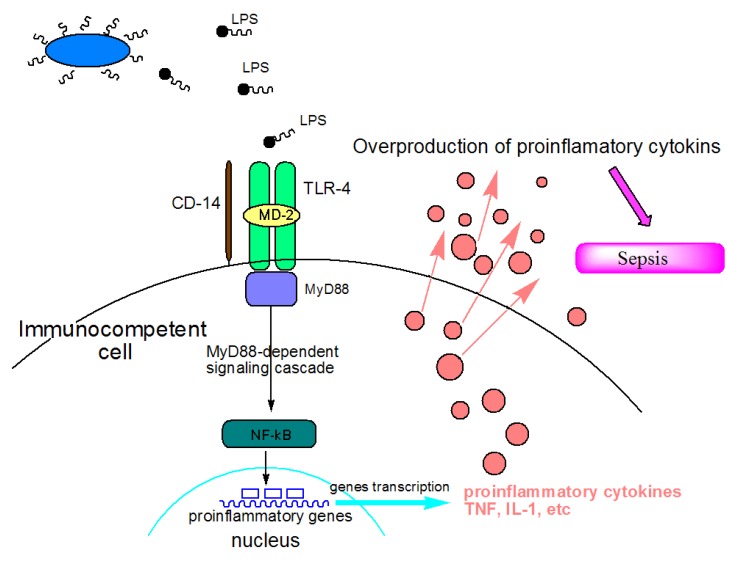
Mechanisms of action of lipopolysaccharide (LPS) as a sepsis inducer. LPS is recognized by Toll-like receptor 4 (TLR-4) on animal’s immune cells. Recognition is aided by two accessory proteins known as CD14 and MD-2. Stimulation of TLR-4 triggers reaction with the adaptor molecule MyD88 (myeloid differentiation primary-response protein 88). The MyD88-depending signaling pathway leads to the activation of nuclear factor-κβ (NF-κβ), which regulates the expression of target genes that encode pro-inflammatory mediators. The overstimulation of receptors may lead to uncontrolled general inflammation and eventually to sepsis.

In recent years, there has been considerable progress in understanding the molecular and cellular mechanisms underlying Gram-negative sepsis and endotoxic shock [2]. These clinical syndromes result from the accumulation of endotoxins (lipopolysaccharide (LPS)) in the blood of the host, which are released from pathogenic bacterial cells during systemic bacterial infections or from the normal bacterial flora within the lumen of the intestine. By interacting with specific receptors on the host effector cells, LPSs induce the synthesis of a large number of endogenous proinflammatory cytokines by these cells. The overproduction of these cytokines leads to an uncontrolled inflammatory reaction, which ultimately manifests into serious physiological disorders in the body (Figure 1).

Therapies for Gram-negative sepsis remain unsatisfactory despite a concerted effort to develop new treatments for this life-threatening syndrome. Currently, no drugs are specific for endotoxin-induced clinical syndromes. The most compelling therapeutic strategy for sepsis would certainly be the elimination of endotoxins so the systematic inflammatory response and the catastrophic cytokine avalanche can be avoided. Hence, compounds with therapeutic potentials as anti-endotoxin agents may include those that either bind LPS at high affinity and neutralize its toxicity or those that competitively interact with LPS receptors on host cells without the activating antagonists receptor. Both groups of agents, acting through different mechanisms, block endotoxin binding to specific receptors on target cells, thus preventing the synthesis of proinflammatory cytokines [2].

## 2. Lipid A, Chitosan and Carrageenan—Marine Compounds with Antiendotoxic Potential

### 2.1. Marine Lipid A Potential Endotoxin Antagonists

**Figure 2:**
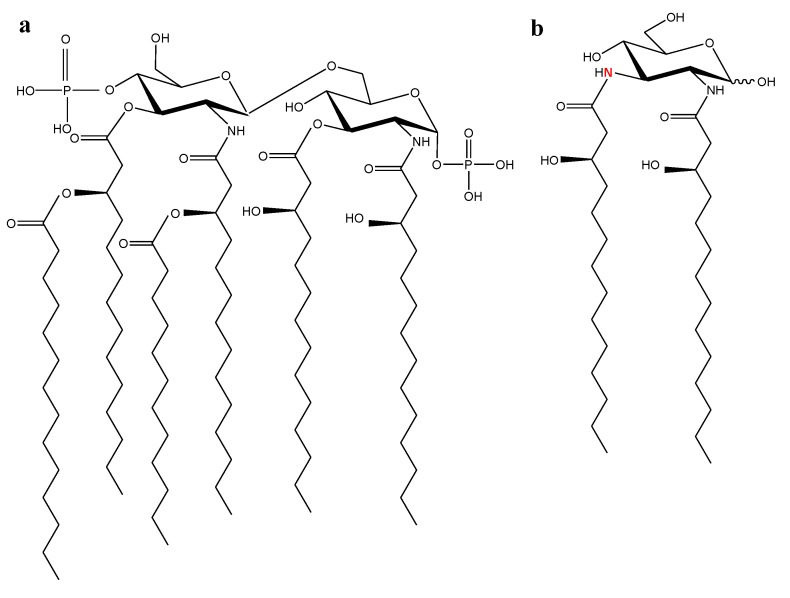
Chemical structure of certain lipid As. (**a**) Lipid A of *E. coli* K-12 [5]; (**b**) Lipid A of *Rhodopseudomonas viridis* [6].

Chemically, LPS consists of a hydrophilic polysaccharide (S-form of LPS) or oligosaccharide (R-form of LPS) covalently linked to a hydrophobic lipid portion designated as lipid A, which is responsible for the inflammatory properties of the endotoxin [2,3]. The most potent lipid A is generally considered to be the hexa-acylated *E. coli* one with acyl residues of 12 to 14 carbons in length and a bisphosphorylated diglucosamine backbone (Figure 2a) [4,5]. Variations in the chemical structure of *E. coli* lipid A, such as changes in the number or length of the acyl chains, the cleavage of a phosphate group, or the replacement of the disaccharide backbone by a monosaccharide backbone (Figure 2b) [6], may lead to a strong decrease in the biological activity [4]. The structural derivatives of lipid A that are capable of inhibiting physiological response triggered by endotoxin not only *in vitro* but also *in vivo*, as demonstrated in animal models of sepsis [7], and could be potentially useful in sepsis therapy [8].

Lipid A derivatives exhibiting anti-endotoxin activities can be obtained via the chemical degradation of the native molecules of LPS or lipid A [9] or as a product of organic synthesis (*i.e.*, synthetic analogues of lipid A [10]). Other sources of endotoxin antagonists based on lipid A include bacterial mutants bearing mutations in the genes involved in lipid A biosynthesis [11] and bacteria that are phylogenetically distant from the family Enterobacteriaceae [12]. We hypothesize that marine bacteria inhabiting specific environmental niches (e.g., low temperature, high hydrostatic pressure, and high salinity) may synthesize lipid A molecules of unusual structures that, which can be possible pharmacological interesting.

**Figure 3:**
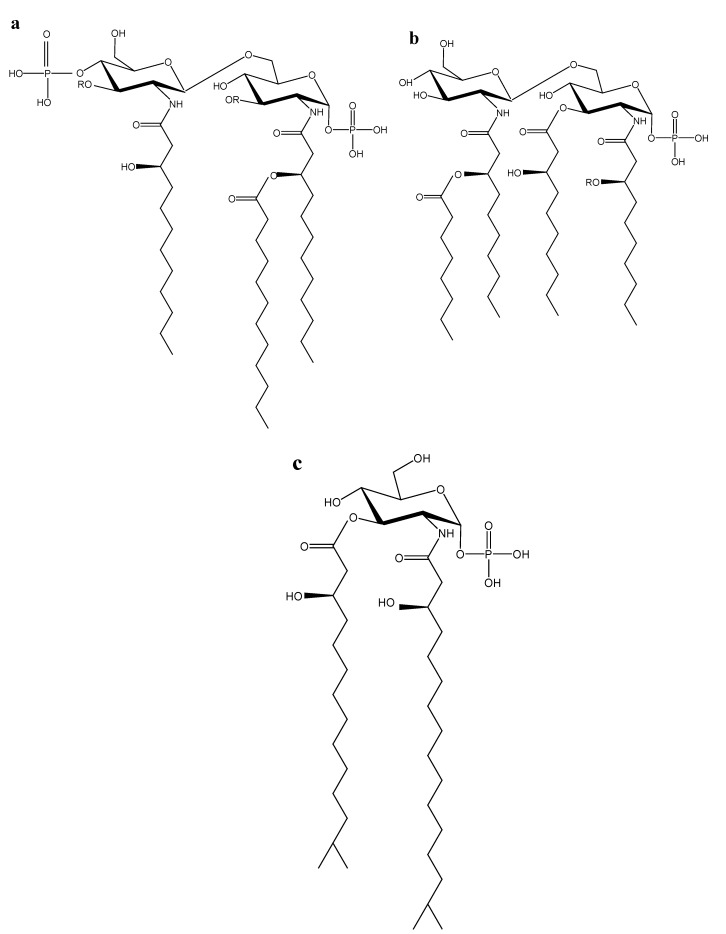
Chemical structure of lipid As from certain marine bacteria. (**a**) Lipid А of *P. haloplanktis* АТСС 14399^Т^ bacteria [17], where R = 3-OH-11:0, 3-OH-12:0, 3-OH-13:0, 3-OH-*iso*-11:0, 3-OH-*iso*-12:0 acid; (**b**) Lipid A of *M. сommunis* ATCC 27118^T^ [21] (I), *M. vaga* ATCC 27119^T^ [20] (II) and *M. mediterranea* АТСС 700492^Т^ [24] (III) bacteria, where (I) −R = C12:0 acid; (II) −R = С10:0, С12:0 or С12:1 acids; (III) −R = 3-ОН-С10:0 acid; С4′ atom in the molecule of this lipid A is replaced with the residue of phosphoric acid; (**c**) Lipid A *C. scophthalmum* CIP 104199^Т^ [22] and *C. indoltheticum* CIP 103168^Т^ [24].

Earlier studies on certain lipid A molecules from marine bacteria only identified their fatty acid components. [13,14]. Later studies revealed a few special features of the lipid A molecules, the most pronounced of which is a penta-acyl type of structure [15]. We conducted systematic studies of lipid A from 13 wild strains of marine Proteobacteria belonging to the families of *Alteromonadaceae* (*Alteromonas*, *Idiomarina*, and *Pseudoalteromonas* genera [16,17,18]) and *Vibrionaceae* (*Shewanella* and *Vibrio* genera [16]) and the genera of *Marinomonas* [19,20,21] and *Chryseobacterium* [22]. The marine lipid As molecules were shown to be characterized by significant structural diversities (Figure 3). Furthermore, these lipid A molecules have a few structural peculiarities: a highly specific fatty acid composition (low content or total absence of normal fatty acids, a presence of *iso*-branched 3-hydroxy fatty acids and, a presence, in some cases, unsaturated fatty acids), a low degree of acylation and phosphorylation, and a monosaccharide backbone (lipid As of the genus *Chryseobacterium*). In addition, unlike the Enterobacteriaceae LPS and lipid As, these molecules have displayed low toxicity in animal models [23].

Due to these findings, biological properties of marine LPSs and lipid As with unusual structures (Figure 3b(I),3b(III),3c) are studied using *ex vivo* bioassays in human whole blood with tumor necrosis factor-α (TNF-α) as a marker of cell activation [24]. TNF-α has been shown to be the most important inflammatory cytokine that mediates multiple immunopathological features of LPS-induced sepsis [23]. Therefore, the ability of the LPS and lipid A to induce and/or inhibit TNF-α release is often used as a reflection of their endotoxic and anti-endotoxic potentials [3].

LPSs and lipid As of the marine bacteria were shown to only weakly induce TNF-α synthesis or not at all in human blood cells. At the same time, these compounds inhibited thexendotoxin-induced production of TNF-α by up to 80%–90% in a dose-dependent manner [15]. As expected, the compounds effectively blocked the TNF-α-inducing activity of any toxic LPS irrespective of its source, its polysaccharide chain length, or its toxicity degree. Their inhibitory activities were dependent on the dose of the endotoxin used as the agonist: for higher doses of endotoxin, greater concentrations of lipid As were needed for efficient inhibition, thus [24] indicating a mechanism of competitive inhibition by the marine lipid As and LPSs that were tested. Interestingly, the LPS from *M. communis* had a much higher inhibitory activity than the well-known synthetic endotoxin antagonist E-5531, which was designed based on the lipid A from *Rhodobacter capsulatus* [25]. The IC_50_ dose of *M. communis* LPS for inhibiting the induction of TNF-α synthesis by 10 ng/mL *E. coli* LPS was approximately 40-fold lower than that of E-5531. Thus, structural and biological characteristics of the marine lipid A molecules that have been studied are similar to those of the lipid A-like molecules that exhibit the properties of endotoxin antagonists. Taken together, the previous results suggest that the search for potential endotoxin antagonists among LPS and lipid A molecules from marine Gram-negative bacteria may be promising.

### 2.2. Chitosan as an LPS Binding and Endotoxin Neutralizing Agent

One of the promising ways to inhibit harmful inflammatory/septic responses of endotoxins is to bind the endotoxins with certain polycations, which can interact with lipid A bearing negatively charged phosphate groups and, as a result, block this toxic center of the endotoxin.

**Figure 4:**
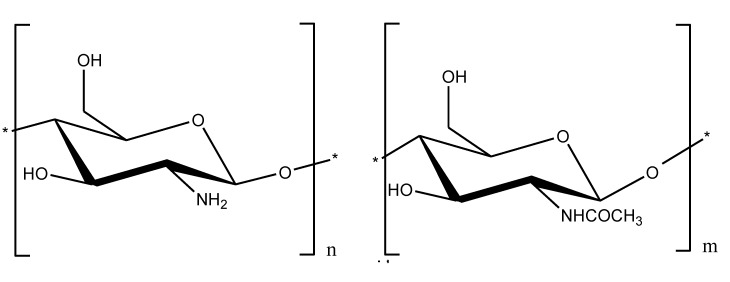
Chemical structure of chitosan.

Chitosan (Ch) (β-1,4-d-glucosaminoglucan) (Figure 4) a polycation that is widely used in many fields, including biomedicine, due to its nontoxicity, biodegradability, biocompatibility and biological activity [26,27]. The ability of chitosan to form specific complexes with polyanions of various natures is well known [28]. Chitosan has been used to clean certain biological fluids as a sorbent with an endotoxin-binding capacity [29]. Moreover, chitosan possesses an antibacterial activity [30] that is usually associated with substances that bind and neutralize endotoxins. Therefore, it would be useful to study chitosan as a potential agent for treating sepsis.

Systematic studies of chitosan binding with LPS and the biological properties of the chitosan-LPS complexes have been conducted. Chitosan has been shown to interact specifically with LPS to form water-soluble stable complexes of various stoichiometry compositions. The apparent binding constant value (*K*_a_) of chitosan with LPS was determined to be approximately 10^5^ M^−1^, which was in line with literature data on the binding constants of LPS complexes with other polycations, such as human serum albumin [31] and NK-lysine [32]. At saturation, there were 9–11 and 200 glucosamine residues per mole of LPS on average in the complexes formed in dilute solutions (LPS concentration <0.1 mg/mL) [33] and in concentrated solutions (LPS concentration 1 mg/mL) [34], respectively. The stoichiometry of chitosan-LPS complexes and the *K*_a_ values were dependent on the reaction conditions, including the temperature, component concentration, pH and ionic strength of solutions. These environmental factors have influenced the LPS aggregate state, illustrating that the LPS aggregate state is the important factor of the interaction between LPS and chitosan [35]. The quantitative parameters of chitosan binding to LPS were also dependent on the structural features of the components and their molecular sizes. An increase in the molecular mass of LPS molecules led to improved affinity between the complex components and a decrease in the LPS content in the complex [33].

The interaction of a cationic polymer such as chitosan with the highly anionic LPS undoubtedly has an electrostatic component involving the ionic binding between negatively charged groups on LPS and positively charged amino groups on chitosan. In a typical LPS molecule, both lipid A and the inner part of the core oligosaccharide carry negatively charged groups [36] that appear to be the major contributors of the net negative charge of the LPS and hence the major binding sites for chitosan. This hypothesis was proposed based on data showing that partial fragments of LPS containing these negatively charged groups could compete with intact LPS for chitosan binding [37]. An essential factor affecting the interaction between chitosan and LPS is pH, which determines the degree of ionization of the charged groups involved in complex formation. It was found that LPS-chitosan complexes are formed in the pH range of 4.0 to 7.0 [38]. The significant role of electrostatic forces in the interaction of endotoxin with chitosan is supported by the fact that the stability of a complex decreases with increasing ionic strength. However, some LPS-chitosan complexes have been shown to maintain their stability in 1 M sodium chloride solutions. This finding clearly indicated the involvement of various types of interactions during complex formation. Based on the carbohydrate nature of these substances, it is believed that, in addition to the electrostatic forces, hydrogen bonds are also involved in the complex formation of LPS with chitosan [38].

The studies have shown that chitosan is a LPS-binding polycation with therapeutic potential. However, it is desirable to increase the efficiency of interaction between chitosan and LPS. It has been demonstrated previously that long-chain aliphatic lipophilic groups, when present in a polycation, could stabilize the complex of the polycation with LPS through hydrophobic interactions between these substituents and acyl lipid A domains [39]. Based on this concept, a. chemical modification of chitosan was performed to increase the affinity of LPS-chitosan binding [37]. The chitosan molecule was modified by a lipophilic substituent (residue of 3-hydroxytetradecanoic acid). The modified chitosans were soluble in aqueous media due to the use of low molecular weight chitosan derivatives and a low degree of their acylation. Using this method, we synthesized mono-acyl-derivatives of chitooligosaccharides (di to tetra) and oligochitosan (5.5 kD) with the acyl substituent positioned at the reducing termini of the molecules [37]. As described previously [39], amphiphilic compounds of this type with distantly localized hydrophobic and hydrophilic regions inside the same molecule interact preferentially with the lipid A fragment, and blocking this interaction is important for the neutralization of LPS [40]. We found that the apparent binding constants of the *N*-acyl derivatives of chitosan (acyl-ch) with LPSs were an order of magnitude higher in comparison with an unsubstituted analogue. The elongation of the carbohydrate chain of the chitosans from 2 to 30 monosaccharide residues also led to an increase in the LPS-binding activity with both substituted and unsubstituted chitosan derivatives [37].

Molecular modeling was used to obtain information about the structure of the LPS-chitosan complexes [37]. The ionic, hydroxyl, and carbonyl groups of the monosaccharide and fatty acid residues in the core and the lipid A moieties of LPSs were demonstrated to be involved in the interaction with chitosans and chitooligosaccharides. The models also showed that the increase in the length of the chitosan carbohydrate chain resulted in multipoint binding on several anionic sites localized in the core and at the lipid A moiety of the LPS molecule, which seemingly led to an increase in the binding affinity. An increase in the amount of acetyl groups in chitosans led to a decrease in the number of bonds stabilizing the complex. In the case of *N*-acylated chitooligosaccharide complexes with LPSs, the fatty acid residues on chitosan were incorporated into the polyacyl domain of lipid A and were located in parallel to the fatty acid chains of lipid A, which reinforced the hydrophobic interactions and increased the affinity of acylated chitosan binding to LPS. These theoretical data for the binding affinity of chitosan [37] to LPS also coincided with the experimental results. [33,34,38]. Thus, water-soluble derivatives of chitosans with hydrophobic substituents and low extent of amino group substitutions are promising compounds for binding to endotoxins.

The incorporation of chitosan and its *N*-acylated derivatives in liposomes can be used to obtain nanoparticles with a high LPS binding capacity. The liposomes covered by chitosan were shown to cause a significant increase in the binding of LPS by the liposomal bilayer. The high efficiency of interaction was achieved due to multipoint binding of LPS with chitosan molecules attached to the liposome surface and additional inclusion of LPS into the phospholipid bilayer. A computer simulation showed that modifying the liposome lipid bilayer with *N*-acylated low molecular weight chitosan increased the binding of LPS with liposomes due to the formation of additional hydrogen and ionic bonds between the molecules of chitosan and LPS [41].

The formation of LPS-chitosan complexes is accompanied by the essential modification of several immunological properties of LPS. As shown previously, LPS-chitosan complexes possess 10–20 times less toxicity than LPS alone [34]. This effect is dependent on the LPS source, the ratio of the components in the complex and the molecular mass of chitosan. A substantial reduction of LPS toxicity in LPS-chitosan complexes may be explained by the blocking of the toxophoric center of endotoxin or the alterations in the molecular charge and/or the structure of LPS aggregates by chitosan [42].

The LPS-chitosan complex has been shown to maintain the ability to induce IL-8 and TNF-α. The induction efficiency of IL-8 and TNF-α by the LPS-chitosan complex, compared with the parent LPS, was found to be approximately 70% for IL-8 and slightly lower for TNF-α [34]. This finding is a possible reason for the lower toxicity of LPS when it is complexed with chitosan because, as mentioned above, the toxic effect of LPS is associated with its ability to stimulate the synthesis of pro-inflammatory cytokines in a host, especially TNF-α. It is known that many LPS-binding cationic peptides and proteins significantly inhibit the ability of LPS to induce cytokines [2]; however, the mechanisms of inhibition have not been established. It is also known that LPS induces the production of cytokines in monocytes and macrophages through TLR-4 receptors [43]. Using human embryonic kidney cells (HEK 293 cells) co-transfected with this receptor and MD2, we showed that the decrease in the ability of LPS within LPS-chitosan complexes to induce the secretion of pro-inflammatory cytokines in macrophages was not dependent on TLR4 and that chitosan did not block the TLR4 receptor binding to LPS and LPS signal transduction in the cell [34]. However, the possibility remains that some degree of inhibition of LPS-TLR4 interaction was caused by chitosan binding to the lipid A portion of LPS. The down-modulation of the cytokine-inducing activity of LPS might also be due to the competition between LPS and chitosan for the receptor CD14. CD14 has been recognized as a common binding receptor on monocytes for both S-LPS and chitosan, and it is involved in the transduction of the cytokine-inducing signal [44].

### 2.3. Chitosan and Carrageenan as Auxiliary Agents for Sepsis Therapy

**Figure 5:**
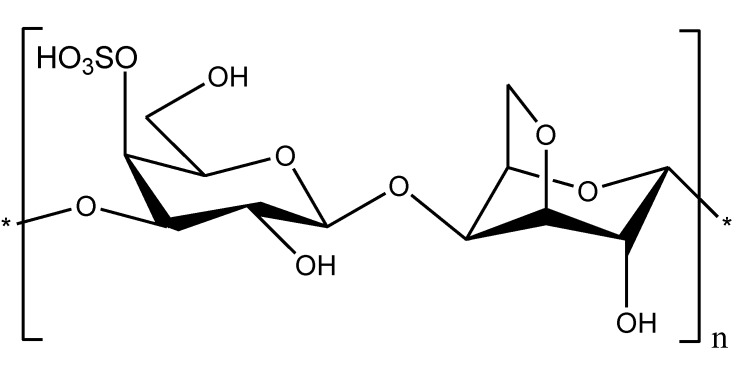
Chemical structure of κ-carrageenan.

Sepsis, which is caused by Gram-negative bacteria, is a complex multifactorial syndrome with inflammatory, procoagulant, and immunosuppressive aspects. Therefore, combination therapies may likely improve current treatment for sepsis [45]. Chitosan and its derivatives may be of particular interest because, in addition to their effects on endotoxins, they have biological properties that may be useful in treating sepsis. Other natural polysaccharides such as carrageenans (Figure 5), which belong to a complex family of sulfated galactans from red marine algae, may be also considered to be potential agents in supporting therapies for sepsis due to their biological activities [46].

Several studies have shown that the immune cells of patients with sepsis are either hyporesponsive or deactivated, suggesting that critically ill patients may be immunosuppressed, especially in the post-acute phase of septic shock [47]. Strategies to boost immunity could improve the outcome of sepsis when applied early, if measures of immune competence indicate impaired immunity, or when applied late in sepsis. Therefore, the use of immunostimulatory substances for the treatment of sepsis is a logical approach [45]. Chitosan and its derivatives as well as carrageenans have been shown to possess potent immunomodulatory activities [48,49]: These compounds stimulated immune cells to produce INF-γ and the anti-inflammatory cytokine interleukin-10.

Coagulation disorders are known to arise during sepsis, and 30%–50% of patients have disseminated intravascular coagulation (DIC), which is the more severe clinical form of sepsis that leads to multiple organ dysfunctions [1]. It has been shown that platelets play an important role in the development of DIC.

The effects of carrageenan and chitosan on donor platelets of healthy subjects and patients with acute alimentary toxico infection with DIC syndrome have been studied. The pretreatment of donor platelets with these polysaccharides reduced LPS-induced platelet aggregation and protected the platelets against ADP, another inducer of platelet aggregation [50,51]. In the patients who received the complex therapy (polysaccharide together with standart medicines), carrageenan actively restored haemostasis and corrected several biochemical indices and parameters of the immune system compound. Conversely, the basic therapy did not yield the level of efficacy in the patients [50].

One significant piece of evidence that supports the potential therapeutic utility of chitosan, its hydrophobic derivatives and carrageenan for treating endotoxemia, sepsis and septic shock is that these polysaccharides provide protection against injected LPS in animal models.

Chitosan, oligochitosan and the *N*-acylated derivatives were shown to protect against LPS-induced mortality in a d-galactosamine-sensitised mouse model. The most protection was observed when the chitosans were administered either simultaneously with LPS or two hours after an LPS challenge [52]. Carrageenans also raised the lethal dose of LPS in experimental animals. Pretreatment with carrageenan significantly prolonged the survival of mice against an LPS challenge [53]. The degree of protection depended on the structures of the carrageenans, their doses, and the routes and times of administration. The best protective effect was observed in mice that were pretreated with κ-carrageenan.

The endotoxin that enters the bloodstream during endotoxemia induced by local or systemic Gram-negative infections, as it is known, has an impact on almost all systems of a macroorganism, causing a number of pathophysiological changes [2,45]. In our experiments, nonlethal doses of LPS that were parenterally injected into the mice caused significant biochemical and pathological changes in the tissues [53,54]. Chitosan and carrageenan at preventive oral administration doses protected mice from endotoxin-induced morphological, endocrine and metabolic disorders in the liver, thymus, spleen and adrenal glands of the animals. These data suggest that these polysaccharides conferred resistance against bacterial endotoxin in the mice. Thus chitosan and carrageenan prevented the physiologically toxic effects of LPS-induced endotoxemia [53,54].

## 3. Conclusions

Marine organisms and microorganisms have long been recognised as a source of novel therapeutics for human diseases. Studies on the potential usefulness of marine compounds in the treatment of endotoxin-induced syndromes suggested that chitosan and its derivatives as well as lipid A from certain marine Gram-negative bacteria could be promising compounds for treating Gram-negative sepsis. In addition, carrageenan and chitosan may be useful for the adjunctive therapy of sepsis due to their anticoagulant activity and immunostimulatory properties, respectively.
